# Features and mechanism of localized enzyme-assisted self-assembly of peptides from unilamellar vesicles

**DOI:** 10.3389/fchem.2026.1800750

**Published:** 2026-03-11

**Authors:** Aymeric Ontani, Jean-Yves Runser, Shahaji H. More, Marc Schmutz, Alain Chaumont, André Schroder, Pierre Schaaf, Loïc Jierry

**Affiliations:** 1 CNRS, Institut Charles Sadron, Université de Strasbourg, Strasbourg, France; 2 INSERM/Université De Strasbourg, UMR_S1121, Strasbourg, France; 3 Faculté de Chimie, Université de Strasbourg, Strasbourg, France; 4 CNRS, INSA Lyon, LaMCoS, Villeurbanne, France

**Keywords:** enzyme-assisted self-assembly, giant/small unilamellar vesicles, molecular dynamic simulation, peptide self-assembly, phospholipid-based membrane

## Abstract

Localized enzyme-assisted self-assembly (LEASA) has emerged as a powerful tool to generate peptide nanofibers from and within bacteria or cancer cell lines. This approach has led to promising developments in medical imaging, antimicrobial treatments and cancer therapies. Despite these achievements, the features of self-assembly processes localized near the plasma cell membranes and induced by enzymes are not easy to study since biological media compositions are complex and vary over time. From model systems based on giant and small unilamellar vesicles (GUV and SUV respectively) displaying phosphatases at the outer edge of their phospholipid’s membrane, we study the self-assembly of peptides triggered by an enzymatic dephosphorylation. Peptide nanofibers, generated in the close vicinity of GUVs, adsorb all around the membrane where enzymes are located. This process induces the formation of a transient permeability through the membrane without destroying the vesicles, as observed by confocal laser scanning microscopy (CLSM). A cryo-transmission electron microscopy (cryo-TEM) monitoring over time highlights a lag-time before the formation of nano-aggregates located all around SUVs, followed by a rapid formation of short nanofibers near or directly from vesicles. Thanks to the presence of enzymes located on the surface of the vesicles, the micrometer-long nanofibers both adhere and exert such a mechanical force on the spherical shape of vesicles that they deform them. Finally, based on classical molecular dynamics simulations, we propose a mechanism that accounts for all our experimental observations, rationalizing the LEASA process induced on the surface of phospholipid bilayers containing phosphatases.

## 1 Introduction

Protein self-assembled fibers are ubiquitous in cells. For example, actin fibers form the framework of the cells by lining their membrane. Microtubules which are a major constituent of mitosis spindles play also a major role during cell division. It is now firmly accepted that all self-assembly processes taking place in cells are spatiotemporally controlled and enzymatically triggered. Enzyme-assisted self-assembly (EASA) processes of short peptide derivatives were introduced by Bing Xu in a seminal work in 2004 (Yang et al., 2004). Because peptides self-assembly takes place in the vicinity of the enzymes, if enzymes are localized, the self-assembly process will also be localized (Muller et al., 2022; Vigier-Carrière et al., 2018). This feature has been exploited within living systems (Liu et al., 2023; Qiao and Xu, 2023; Shi and Xu, 2015; Shy et al., 2019). By using suitable phosphorylated precursor peptides, self-assembly resulting into nanofibers has been induced around and inside bacteria or cancer cell lines, that express high concentrations of phosphatase on their membrane, for *in vitro* microscopy imaging or efficient therapeutic developments (Feng et al., 2019; Kuang et al., 2014; Li et al., 2013; Pires et al., 2015; Zhou et al., 2016). The application of the Localized EASA (LEASA) concept to living systems has also proven effective in the case of enzymes other than phosphatases (Carlini et al., 2019; Jiang et al., 2017; Zhou et al., 2015). In the quest of synthetic cells, enzyme triggered and spatially controlled self-assembly processes appeared as an interesting tool. However, long before the application of peptides LEASA to the surface of plasma membranes, this approach was studied at the solid-liquid interface. Indeed, in 2009 Ulijn’s group grafted enzymes on a solid surface and observed the formation of small fibers on the surface when it was brought in contact with peptide precursors (Williams et al., 2009). This was the first example of synthetic LEASA. Later our groups extensively worked on LEASA processes by embedding enzymes on surfaces through polyelectrolyte multilayers or by localizing the enzymes within gels. Different types of enzymes so deposited on surfaces were investigated and have resulted in the formation of a dense “forest” of fibers which are more or less oriented perpendicularly to the surface (Rodon Fores et al., 2019; Rodon Fores et al., 2020; Rodon Fores et al., 2021; Vigier-Carrière et al., 2015; Vigier-Carrière et al., 2017). This has also been observed by others (Guo et al., 2024). When incorporating enzymes in gels, we observed the formation of reaction-diffusion like patterns of peptide self-assembled areas (Runser et al., 2022; Runser et al., 2023; Runser et al., 2024). Thus, the spatial localization of enzymes is not trivial in the process of self-assembly and growth of the fibrillar network.

The current picture of the peptide self-assembly mechanism has been long to emerge due to the experimental difficulties to grasp the crucial initial stages of the process. It is now accepted that the peptide self-assembly takes place in several steps following Ostwald’s step rule (Jiang et al., 2021; Levin et al., 2014). The slow initial stage is the formation of unordered highly concentrated liquid droplets often termed coacervates (Yuan et al., 2019). In these coacervate droplets, the high local hydrogelator concentration and their mobility favours the formation of small ordered self-assembled clusters, some of them reaching a critical size to form a critical self-assembly nucleus. This is the second step of the self-assembly process, faster than the initial stage. Once the critical nucleus has formed, the fiber grows rapidly by aggregating hydrogelator molecules on its ends. Very recently, a full pictures could be obtained from simulations, showing that the fiber growth process occurs merely by adsorption of hydrogelators on the fibers, diffusion of them along the fibers followed by their capture at the fiber ends (Piskorz et al., 2025). It is also suggested that at high concentration, the hydrogelators adsorbed on a fiber form along it unordered aggregates from which results “secondary” fiber formation. Even if for LEASA processes more or less similar mechanism pathways are anticipated, it has been shown that proteins not only transform precursors into hydrogelators, but they also play a role in the induction of the self-assembly process itself and its resulting morphology or chirality (More et al., 2025).

Here we present a first LEASA process of peptides on giant and small unilamellar vesicles (GUVs and SUVs respectively) through the trigger of enzymes anchored on the vesicle bilayers. We will use both confocal microscopy and cryo-TEM to observe the initial stages of the self-assembly process allowing to visualize some of the anticipated initial processes and their impact on the vesicles. Molecular dynamic investigations will be carried out to rationalize a mechanistic pathway of LEASA of small peptides from phospholipid bilayers.

## 2 Results and discussion

**FIGURE 1:**
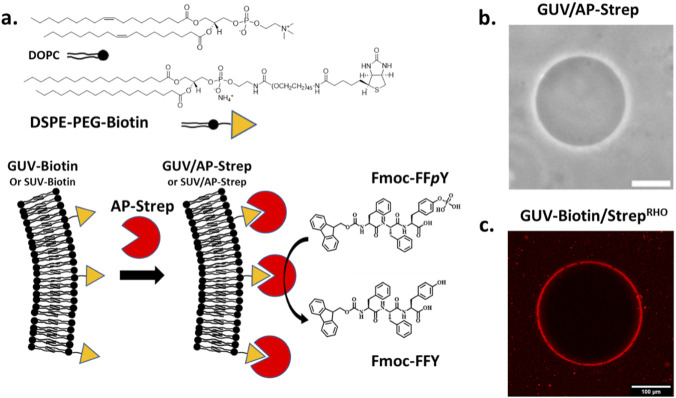
**(a)** Molecular structure of the phospholipids involved in the GUV (or SUV) preparation, and Fmoc-FF*p*Y/Fmoc-FFY model couple of precursor/self-assembling peptide for LEASA induced by AP-Strep. Typical phase-contrast microscopy and epifluorescence microcopy images of **(b)** GUV/AP-Strep and **(c)** GUV/AP-Strep^RHO^, respectively. The white bar corresponds to 100 µm.

An alkaline phosphatase (AP) was introduced at the outer edge of a phospholipid bilayer of GUV to trigger the self-assembly of a tripeptide Fmoc-FFY through the enzymatic hydrolysis of the phosphorylated precursor Fmoc-FF*p*Y (Fmoc, fluorenyloxymethoxycarbonyl; F, phenylalanine; Y, tyrosine, *p*Y: O-phosphorylated tyrosine). This tripeptide model Fmoc-FFY is effective for LEASA (Figure 1a) (Rodon Fores et al., 2021; Vigier-Carrière et al., 2015; Wang et al., 2014). Indeed, when using 1 mg/mL concentration of Fmoc-FF*p*Y (or lower), this phosphorylated precursor does not lead to nanofiber formation. In these conditions, only the dephosphorylated Fmoc-FFY tripeptide self-assembles resulting in a nanofibrous network. The phospholipid bilayer based vesicles were formed from a mixture of 1,2-dioleoyl-sn-glycero-3-phosphocholine (DOPC) and 1,2-distearoyl-sn-glycero-3-phosphoethanolamine-N-[biotinyl (polyethylene glycol)-2000] (DSPE-PEG-Biotin), inspired from reported methods (Weinberger et al., 2013). Details of the GUV preparation are given in Part 1.2 of the Supplementary Material (SM). These GUVs containing 1% of biotin-derived phospholipid, called GUV-Biotin, are brought in contact with alkaline phosphatase-streptavidin bioconjugate (AP-Strep) in order to anchor the enzyme on the vesicle membrane through a biotin-streptavidin interaction. Advantageously, the resulting vesicles called GUV/AP-Strep, were prepared in a sucrose solution and once formed, they were transferred into a glucose solution for the rest of our study. Due to the density difference between glucose and sucrose, the vesicles can easily be visualized in contrast phase microscopy, showing GUV/AP-Strep with roughly 10–25 µm in diameter although some vesicles with much larger diameters can also be found (Figure 1b). Size distributions of all vesicles so prepared are given in Supplementary Figure S1 (in SM). The vesicles GUV-Biotin or GUV/AP-Strep remained stable for weeks and using streptavidin labelled with rhodamine (Strep^RHO^), we confirmed by epifluorescence microscopy that proteins are homogeneously distributed all around the GUV-Biotin/Strep^RHO^ surface (Figure 1c).

**FIGURE 2:**
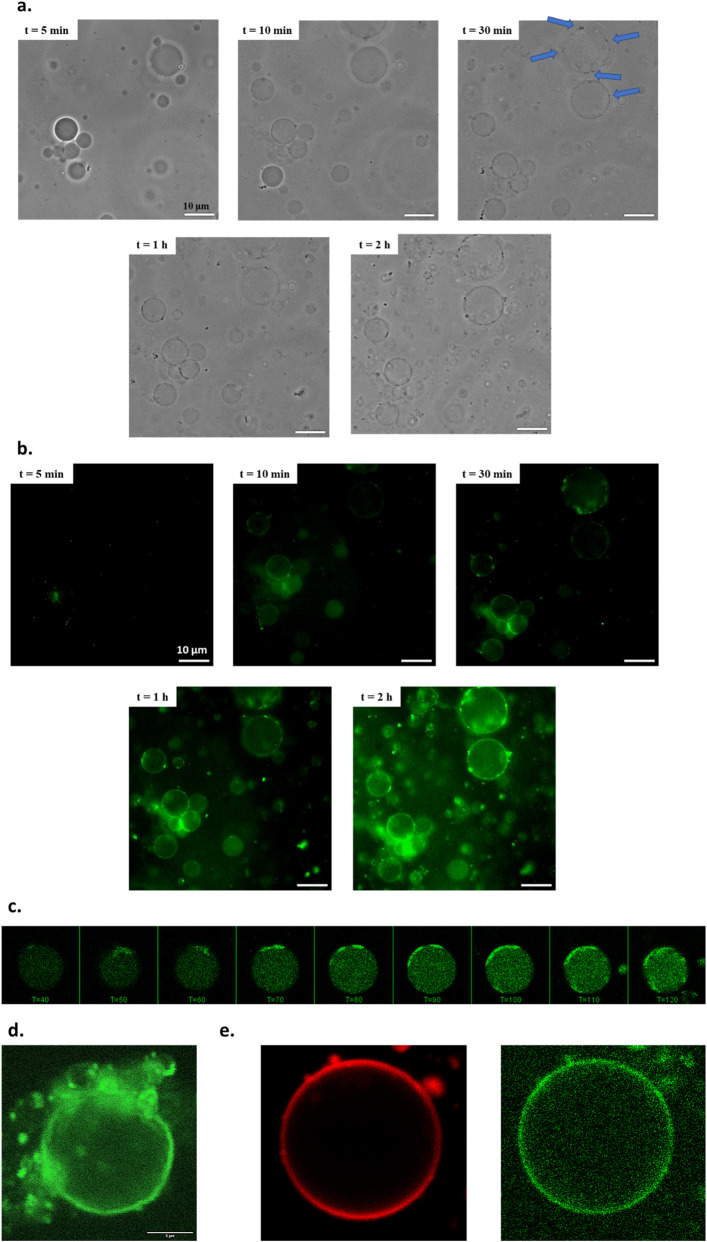
Typical images of GUV/AP-Strep observed overtime after the introduction of Fmoc-FF*p*Y (0.1 mg/mL, t = 0 min) by **(a)** phase contrast microscopy, **(b)** epifluorescence microscopy and **(c)** CLSM in presence of ThT (0.15 mg/mL) introduced concomitantly with Fmoc-FF*p*Y (λ_em_ = 590 nm; λ_ex_ = 457 nm) at t = 0 min. Blue arrows point to the appearance of black dots. **(d)** CLSM image of GUV/AP-Strep, 24 h after the introduction of both Fmoc-FF*p*Y and ThT. **(e)** CLSM images of GUV/AP-Strep^RHO^ observed 2 h after the introduction of both Fmoc-FF*p*Y and ThT: (left) excitation at 561 nm allows to detect the fluorescence emission (in red) of rhodamine-labelled AP-Strep^RHO^; (right) excitation at 458 nm allows to detect the ThT flurorescence emission of Fmoc-FFY self-assembly.

Then, we performed phase-contrast microscopy monitoring over 2 h to visualize the impact of the peptide self-assembly induced near the surface of **GUV/AP-Strep**. When brought in contact with a solution of Fmoc-FF*p*Y (0.1 mg/mL), a latent period of 10 min is observed (in our experimental conditions), during which we note no significant changes (Figure 2a). Then, small black dots appear on the surface of the vesicles, their number increasing over time. This dot formation does not lead to a homogeneous distribution on each vesicle, but these dots seem to stick to each other leading both to localized areas of high dot density on the surface of the vesicles, and other areas without dots. Concomitantly, the phase contrast between the inside of vesicle and their surroundings vanishes over time. Thus, the presence of the precursor peptide Fmoc-FF*p*Y at 0.1 mg/mL concentration results in a transient permeability of the GUV/AP-Strep membrane. When a ten times higher concentration of Fmoc-FF*p*Y (*i.e.* 1 mg/mL) is used, small dots are observed earlier but after 10 min, all GUV/AP-Strep have disappeared, confirming a relation between the bilayer permeability and the peptide assembly. In addition, in the absence of Fmoc-FF*p*Y or when using GUV-Biotin in presence of Fmoc-FF*p*Y, no dots and no loss of phase contrast are observed even after 24 h. Furthermore, when Fmoc-FFY self-assembled nanofibers are brought in contact with GUV-biotin, all vesicles keep their integrity and no significant changes of their phase contrast are noted (see Supplementary Figure S2 in SM). To observe the nanofibrillar network formation based on Fmoc-FFY self-assembly, Thioflavine T (ThT), a specific dye revealing β-sheet structures of Fmoc-FFY, has been introduced concomitantly to Fmoc-FF*p*Y. Using the epifluorescence microscopy mode, we distinguished the shape of GUV/AP-Strep which becomes light green 10 min after the addition of Fmoc-FF*p*Y (Figure 2b). This lag time is quite surprising since the dephosphorylation of the precursor peptide in Fmoc-FFY by AP in solution is a rapid enzymatic reaction, as reported in the literature, meaning that the self-assembly takes time (Bigo-Simon et al., 2023). After more than 30 min, strong fluorescent green spots are visible on almost all GUV/AP-Strep. Remarkedly, these green dots correspond strictly to the black dots visible in contrast phase microscopy suggesting that these dots contain high density of self-assembled Fmoc-FFY nanofibers. Furthermore, we also noticed that when several dots are close to each other, on the same GUV/AP-Strep, or when several GUV/AP-Strep exhibiting these dots are in close proximity, the environment all around them is also significantly greener. Therefore, the presence of dots and the formation of the Fmoc-FFY self-assembled nanofibrous network are two correlated phenomena. The same time-monitoring study was also done by CLSM confirming the observations described above (Figure 2c). However, CLSM allows for a better examination of the phospholipid bilayer of GUV/AP-Strep during the assembly process occurring on its surface and in its surrounding. This monitoring reveals that the vesicles shrink slightly over time and its external aspect appears really greener and rougher, 2 h after the introduction of Fmoc-FF*p*Y (Figure 2d). This is indicating that the peptide self-assembly is initiated at the surface of the vesicles and the resulting self-assembled nanostructures remain localized along the GUV/AP-Strep and disturbs their membrane which becomes rough. We can expect that this perturbation could render the vesicle membrane transiently permeable resulting in a loss of contrast between the vesicles and their surroundings, as observed. It should be noted that the addition of ThT concomitantly with Fmoc-FF*p*Y or just before fluorescence emission images taken, results in similar observations obtained from epifluorescence microscopy or CLSM, showing the absence of impact of this dye in the localized self-assembly process (Tikhonova et al., 2021). Using both AP-Strep^RHO^ to prepare GUV/AP-Strep^RHO^ and ThT, CLSM shows unambiguously a concomitant fluorescence emission in both green and red located at the dots appearing overtime at specific spatial areas of the vesicle membrane (Figure 2e). Thus, in spite of an homogenous enzyme distribution observed before the introduction of the phosphorylated precursor Fmoc-FF*p*Y (Figure 1c), but because all phospholipids move in the fluid bilayer, the formation of dots results from a local concentration of both peptide self-assembled nanostructures and enzymes. In addition, these clusters are growing over time, which confirms the interaction of AP and the self-assembled peptide nanofibers. Their localized over-concentration at certain points around GUV/AP-Strep explains why it is in this confined environment that we observe a higher density of fibrous peptide networks.

**FIGURE 3:**
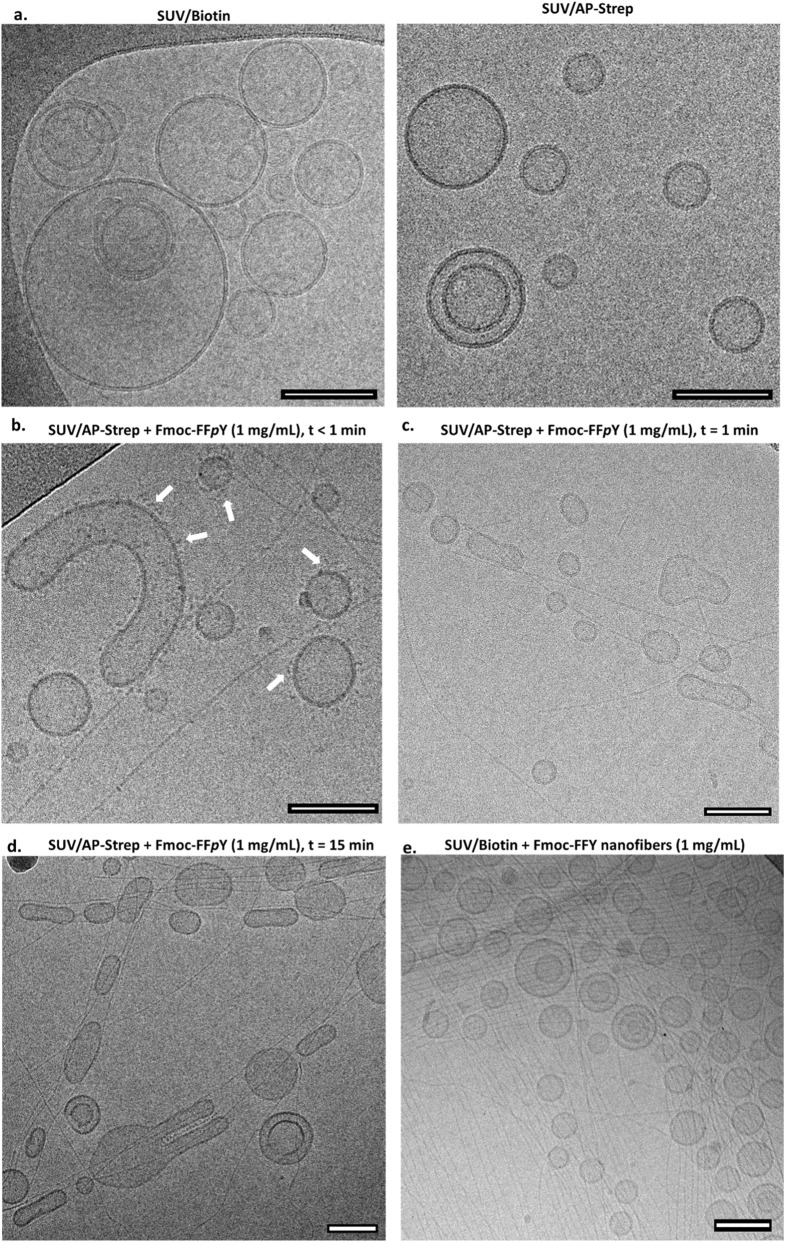
Typical cryo-TEM images of **(a)** SUV/Biotin (left) and SUV/AP-Strep (right), SUV/AP-Strep in presence of Fmoc-FF*p*Y observed **(b)** less than 1 min, **(c)** after 1 min and **(d)** 15 min later. The white arrows show the presence of small aggregates. **(e)** SUV/Biotin brought in contact during 24 h with Fmoc-FFY self-assembled nanofibers formed by annealling (More et al., 2024). The error bar indicates a length of 100 nm.

In order to visualize the nanofiber formation from the vesicles, we observed them by cryo-transmission electron microscopy (cryo-TEM). This is possible by using small unilamellar vesicles (SUV) whose size are in the 100 nm range instead of GUV. These SUVs were prepared from 10% DSPE-PEG-Biotin incorporated during the DOPC-based vesicle formation, leading so to SUV-Biotin. Then, SUV/Biotin were brought in contact with AP-Strep and the excess of the conjugated enzyme was removed through a suitable chromatographic method (see Part 2 in SM). In the absence of Fmoc-FF*p*Y, the resulting SUV/AP-Strep are perfectly rounded-shaped and have a fully rough surface compared to SUV/Biotin. This observed roughness is in agreement with the presence of the AP-Strep and its homogeneous distribution all over the SUV/AP-Strep surface (Figure 3a). When SUV/AP-Strep are brought in contact with a high Fmoc-FF*p*Y peptide concentration (10 mg/mL), self-assembled Fmoc-FFY nanofibers having 5 nm diameter, appear all over the substrate and one cannot distinguish the origin of the fibers (Supplementary Figure S3). To observe the initial stages of the self-assembly process, we worked with lower precursor peptide Fmoc-FF*p*Y concentrations (1 mg/mL) and quenched the sample shortly after contact of SUV/AP-Strep. At the initial stages, *i.e.* immediately after the addition of Fmoc-FF*p*Y to SUV/AP-Strep, one also observes the presence of small dots both on the vesicle’s surfaces and along the fibers (Figure 3b). These dots are not present in the absence of peptides and therefore, they must be attributed to small peptides self-assemblies. One also observes small aggregates close to a vesicle at a location where starts a self-assembled nanofiber. These indicate that the nanofibers emerge from these aggregates, as predicted by simulations (Piskorz et al., 2025). Many SUV/AP-Strep are also decorated by such aggregates. At later stages of the process (≈15 min after the Fmoc-FF*p*Y addition), aggregates are no longer present, neither on the vesicles nor on the nanofibers. We observe that some Fmoc-FFY nanofibers originate from SUV/AP-Strep and some are tangent to the vesicles (Figure 3c). One also observes that many of the vesicles are elongated with the nanofibers being tangent to them (Figure 3d). This seems to indicate a strong interaction between the fibers and the vesicles. When SUV/Biotin are brought in contact with self-assembled Fmoc-FFY nanofibers formed by annealing, (More et al., 2024) thus in absence of AP, SUV/Biotin and nanofibers appear totally independent and the vesicles keep their round-shape morphology (Figure 3e). Thus, this negative control experiment highlights the crucial role of the enzyme in the interaction between the vesicle’s membrane and the peptide self-assembled nanofibrous network. The reason why the SUV/AP-Strep adopt elongated shape could be the following: when the SUV/AP-Strep solution and the Fmoc-FFpY precursor peptide are deposited on the holey carbon film TEM grid, the *in situ* generated self-assembled Fmoc-FFY nanofibers adsorb onto the carbon surface around the holes. Because of the presence of these holes in the supporting carbon film, the mixture solution flows through them, creating local shear stresses that can partially orient the nanofibers as they are blocked on the carbon film. When vesicles expose phosphatase on their surface, such as SUV/AP-Strep, these nanofibers interact with the vesicles through enzyme–nanofiber interactions. Thus, when these nanofibers adsorb onto the surface of the carbon film, the vesicles already bound to the fibers undergo the same shear stresses and take on an elliptic and elongated shape (Figure 3d) (Arraud et al., 2014). This phenomenon is not observed when Fmoc-FFY fibers are placed in contact with vesicles lacking enzymes, such as SUV/Biotin, because this latter cannot interact with the nanofibers (Figure 3e).

**FIGURE 4:**
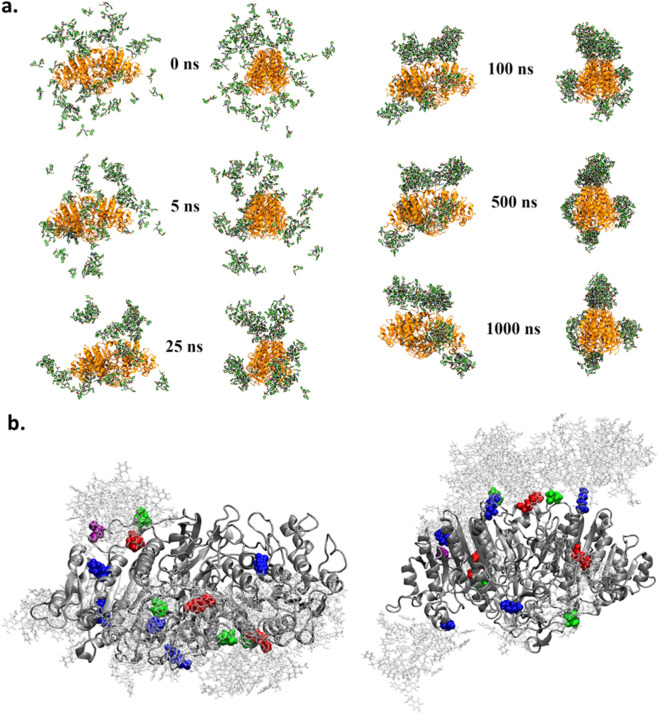
Molecular dynamic simulations showing **(a)** a time-lapse evolution over 1,000 ns of 75 Fmoc-FFY in presence of 1 AP in an icosahedral box. **(b)** (*left*) Instantaneous view of AP, with residues located within 2 Å of an Fmoc-FFY shown in color (LYS residues in blue, ARG in red, ASN in yellow, THR in green, TRP in cyan, and ASP in purple). (*right*) Same view as on the *left*, but rotated by 90°.

To further support these observations, we performed molecular dynamic simulations on a system composed of one AP in aqueous solution surrounded by 75 randomly distributed Fmoc-FFY peptides within an icosahedral box (Figure 4a, see Part 1.7 in SM). Since the enzyme rapidly converts Fmoc-FF*p*Y into Fmoc-FFY, this setup represents the initial stage in which the self-assembling Fmoc-FFY building blocks accumulated near the enzyme. The total simulation time was 1,000 ns. At the beginning of the trajectory, several Fmoc-FFY peptides spontaneously interacted to form dimers or trimers. Within the first 5 ns, additional peptides bound to the enzyme surface, while peptide aggregates also formed in the bulk solution, both in an irreversible manner. After 25 ns, Fmoc-FFY aggregates in solution are recognized by Fmoc-FFY peptides already adsorbed on the AP surface. By 100 ns, no free Fmoc-FFY monomers or aggregates remained in the bulk phase. Notably, only specific regions of the enzyme surface interacted with the peptides, while others remained free peptides. This preferential adsorption led to localization of peptide aggregation on specific areas of enzymes and the subsequent growth of nanostructures extending outward from the enzyme. At least up to 1,000 ns, these aggregates of peptides keep their position on AP, but their internal structural organization continues to evolve. The interaction between Fmoc-FFY and AP appears to be driven by (*i*) electrostatic interaction between the peptide’s C-terminal carboxylic acid and mainly the positively charged lysine side chains of the enzyme, and (*ii*) by hydrogen bonds. Throughout the last 100 ns of the simulation, the number of hydrogen bonds between the AP and the various peptides stabilized at an average of 15.0 ± 3.0 (Supplementary Figure S4). These interactions primarily involve lysine residues, and to a lesser extent arginine, asparagine and glutamine residues on AP, playing mainly the role of hydrogen donor, with the tyrosine residue on the Fmoc-FFY peptide (Figure 4b; Supplementary Figure S5). Overall, these results provide a molecular-level explanation for the formation of nanofibers on enzyme-modified vesicles, showing that the nucleation of the self-assembly process originates from the enzyme surface. In addition, it shows that the Fmoc-FFY self-assembly occurs initially at specific areas on AP and we can thus expect that the interaction between the resulting peptide nanofibers and the enzyme remains, as already reported (More et al., 2024). This may account for both the formation of peptide nanofibers in close vicinity to AP-modified vesicles and the adhesion and deformation of **SUV/AP-Strep** along the nanofibers.

**FIGURE 5:**
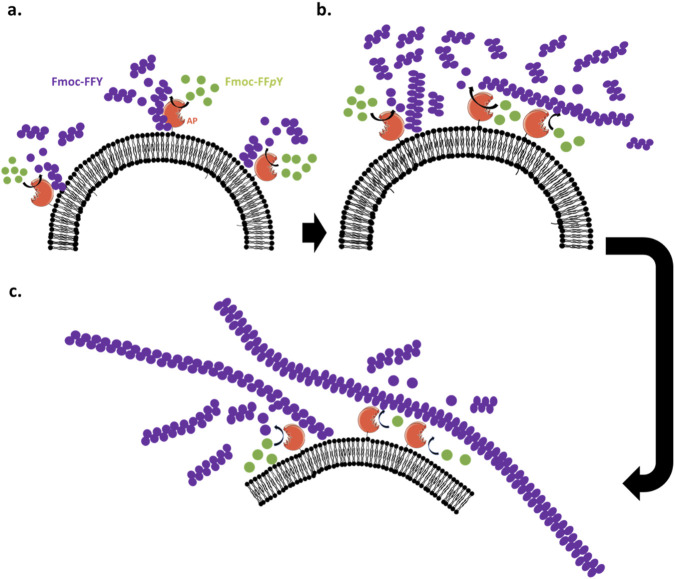
Three steps **(a-c)** scheme of the expected LEASA mechanism occurring from the surface of phospholipid vesicles modified by AP.

Based on our experimental observations and simulation results, we can propose the following mechanism of the LEASA process on phospholipid vesicles (Figure 5). The enzyme (*i.e.* AP, truncated orange) transforms the phosphorylated precursors Fmoc-FF*p*Y (green dots) into self-assembling peptides Fmoc-FFY (purple dots). These self-assemble and then accumulate in an environment close to the membrane. Some Fmoc-FFY and resulting aggregates (purple grape cluster) interact with the surface of the enzyme (Figure 5a). It thus explains the high fluorescence (ThT emission highlighting the peptide self-assembly) observed both in close vicinity of GUV/AP-Strep and all along their membrane. Because the phospholipid membrane is a two dimensional fluid, the APs linked to the phospholipids diffuse and be « captured » by the peptide nanofibers with which they ineract (Figure 5b). This results in a local accumulation of AP at specific areas on the vesicle surface membranes where thus high production of self-assembling and aggregated peptides occur. Experimentally, this leads to highly fluorescent « dots » located at specific areas on GUVs, as observed by CLSM. Moreover, interaction of AP with longer peptide nanofibers seems to have two consequences on the vesicles: first, it induces a mechanical force on the phospholipid membrane which can generate a transient permeability, revealed by a loss of phase-contrast observed over time on GUV/AP-Strep when brought in contact with Fmoc-FF*p*Y. This results finally in vesicles not perfectly round-shaped (Figure 5c). Secondly, because enzymes also act as anchoring points for peptide nanofibres, phospholipid vesicles, which are highly flexible entities, can spread out and elongate along the nanofibres thanks to enzyme–peptide based nanofiber interactions, as observed by cryo-TEM on SUV/AP-Strep.

## 3 Conclusion

As reported in the literature, LEASA on the surface of living systems (cells, bacteria) represents an emerging strategy of interest for biomedical applications (Carlini et al., 2019; Feng et al., 2019; Jiang et al., 2017; Kuang et al., 2014; Li et al., 2013; Pires et al., 2015; Zhou et al., 2015; Zhou et al., 2016). Our work illustrates for the first time the possibility of studying the impact of peptide self-assembly induced by enzymes present on the surface of phospholipid vesicles, using a simple model system. This enabled us to demonstrate that the resulting self-assembled peptide nanofibres interact with the membranes of the vesicles, exerting a mechanical force capable of inducing transient permeability and deforming their spherical shape. The interaction between the nanofibres and the vesicles is ensured by the presence of enzymes that act as anchor points, mainly through hydrogen bonds and electrostatic interactions.

This model system can be refined or optimized by introducing different membrane proteins and/or other phospholipids. Other types of enzymes conjugated to streptavidin could be studied, making this model particularly versatile. These adaptations can be used to study the LEASA phenomenon in a simple system, thereby helping to understand impacts on living cells.

## Data Availability

The raw data supporting the conclusions of this article will be made available by the authors, without undue reservation.
