# Supplementary material for: Features and mechanism of localized enzyme-assisted self-assembly of peptides from unilamellar vesicles
Source: Front Chem. 2026 Mar 11;14:1800750. doi: 10.3389/fchem.2026.1800750 (PMC13013439; doi:10.3389/fchem.2026.1800750)
Supplement: Supplementary file 1 [file Supplementaryfile1.docx]

Supplementary Material

Features and Mechanism of Localized Enzyme-Assisted Self-Assembly of Peptides from Unilamellar Vesicles

Aymeric Ontani,^1^ Jean-Yves Runser,^1,2^ Shahaji H. More,^1,2^ Marc Schmutz,*^1^ Alain Chaumont,^3^ André Schroder,^1,4^ Pierre Schaaf*^2^ and Loïc Jierry*^1^

^1^Université de Strasbourg, CNRS, Institut Charles Sadron, UPR22, 23 rue du Loess, BP 84047, 67034 Strasbourg Cedex 2, France.

^2^ INSERM/Université de Strasbourg, UMR_S1121, Strasbourg, France.

^3^Université de Strasbourg, Faculté de Chimie, UMR7140, 1 rue Blaise Pascal, 67008 Strasbourg Cedex, France.

^4^CNRS, INSA Lyon, LaMCoS, UMR5259, 69621 Villeurbanne, France.

| **1.** | **Supplementary Data** |  |
| --- | --- | --- |
|  | - 1. List of chemicals and abbreviations…………………………………………………. | 2 |
|  | - - 1. Rhodamine B streptavidin or AP-Strep conjugate……………………………….. | 4 |
|  | - 1. Preparation of **GUV**…………………………………………………………………. | 4 |
|  | 1.2.1. MilliQ Water………………………………………………………………………. | 4 |
|  | 1.2.2. Preparation of 5% PVA solution…………………………………………………... | 4 |
|  | - - 1. Preparation of borax buffer, sucrose and glucose solutions……………………… | 4 |
|  | - - 1. Film hydration method to get the **GUV**………………………………………….. | 4 |
|  | - 1. Preparation of **SUV**………………………………………………………………… | 5 |
|  | 1.3.1. Sonication extrusion method………………………………………………………. | 5 |
|  | - - 1. Addition of AP-Strep and Sepharose column purification of **SUV/AP-Strep**……. | 5 |
|  | - 1. Phase-contrast and epifluorescence microscopy…………………………………… | 6 |
|  | - 1. Confocal laser scanning microscopy……………………………………………….. | 6 |
|  | - 1. Transmission electron microscopy in cryo mode…………………………………… | 7 |
|  | - 1. Molecular dynamic simulations…………………………………………………….. | 7 |
|  |  |  |
| **2.** | **Supplementary Figures and Tables** |  |
|  | 2.1. Supplementary Figures……………………………………………………………… | 9 |
|  | Figure S1………………………………………………………………………………… | 9 |
|  | Figure S2…………………………………………………………………………… …… | 10 |
|  | Figure S3………………………………………………………………………………… | 11 |
|  | Figure S4………………………………………………………………………………… | 12 |

# Supplementary Data

- 1. **List of chemicals and abbreviations**

| **Name, acronym, structure** | **M_w_**  **(g.mol^-1^)** | **Supplier** | **CAS number** |
| --- | --- | --- | --- |
| 1,2-dioleoyl-sn-glycero-3-phosphocholine, **DOPC**   | 786.113 | Avanti Polar Lipids | 4235-95-4 |
| 1,2-distearoyl-sn-glycero-3-phosphoethanolamine-N-[biotinyl(polyethylene glycol)-2000] (ammonium salt),  **DSPE-PEG-Biotin**   | 3014.867 | Avanti Polar Lipids | 385437-57-0 |
| 1,2-dipalmitoyl-sn-glycero-3-phosphoethanolamine-N-(lissamine rhodamine B sulfonyl) (ammonium salt),  **16:0 Liss Rhod PE**   | 1248.681 | Avanti Polar Lipids | 384833-01-6 |
| Sucrose | 342.30 | Sigma Aldrich | 57-50-1 |
| Glucose | 180.16 | Alfa Aesar | 50-99-7 |
| Polyvinyl alcohol, **PVA** | 11000 - 31000 | Alfa Aesar | 9002-89-5 |
| Sodium tetraborate, borax | 201.22 | Sigma Aldrich | 1330-43-4 |
| Fmoc-Phe-Phe-Tyr(PO_3_H_2_)-OH, **FmocFF*p*Y**   | 777.54 | Prepared from Rodon Fores et *al*. *Polymers* **2021**, *13*, 1793. | - |
| Thioflavin T, **ThT**   | 318.86 | Sigma Aldrich | 2390-54-7 |
| Phosphatase, Alkaline from bovine intestinal mucosa, **ALP/AP** | ~ 160 kDa | Sigma Aldrich | 9001-78-9 |
| Streptavidin-Alkaline Phosphatase from *Streptomyces avidinii*, **AP-strep**  1 mg solubilized with borax (1 mL) and used as such | ~ 218 kDa | Sigma Aldrich | - |
| Sepharose® CL-4B | - | Sigma Aldrich | 61970-08-9 |
| Chloroform | 119.38 | VWR | 200-663-8 |
| Rhodamine B isocyanate | 536.06 | Sigma Aldrich | 36877-69-7 |
| Streptavidin from *Streptomyces avidinii* | ~58 kDa | TCI Chemicals | 9013-20-1 |
| Sodium carbonate, Na_2_CO_3_ | 105.99 | Alfa Aesar | 497-19-8 |
| Sodium chloride, NaCl | 58.44 | Fischer Scientific | 7647-14-5 |
| Dimethyl sulfoxide, DMSO | 78.13 | Fischer Scientific | 67-68-5 |
| Dialysis membrane | 3 500 | Carl ROTH | - |

- - 1. **Rhodamine B streptavidin or AP-Strep conjugate**

Streptavidin (0.360 mg) was added to a Na_2_CO_3_ aqueous solution (0.400 µL, 0.1 mol.L^-1^). A solution of rhodamine B isocyanate (5 mg) in DMSO (2.5 mL) was added to the previous solution. Reaction was stirred at 4 °C for 4 hours. Dialysis was carried out in 35 kDa MWCO membrane first for 2 hours in H_2_O, then overnight and 7 hours in aqueous NaCl (1 mol.L^-1^), and again with H_2_O for several days, water was changed a few times. Dialyzed solution was lyophilized for 24 hours. Obtained purple powder was stored at 4 °C. This protocol is inspired from Mertz et *al*. *Nat. Mater.* **2009**, *8*, 731–735.

**1.2. Preparation of GUV**

**1.2.1. MilliQ Water**

The ultrapure or MilliQ water with a resistivity of 18.2 MOhm・cm, used for the preparation of all suspensions, solutions, and samples, was obtained with a MilliQ Advantage A10 water purification system from Merck-Millipore (Molsheim, France).

**1.2.2. Preparation of 5% PVA solution**

PVA (50 mg) was added in MilliQ (9.5 mL). This suspension was heated up to 90 °C until complete dissolution of the solid PVA. The resulting homogeneous solution was then allowed to cool down to room temperature (roughly 23°C) before use.

- - 1. **Preparation of borax buffer, sucrose and glucose solutions**

25 mM borax buffer was prepared by dissolving sodium tetraborate (2.5 g) in fresh MilliQ water (500 mL), and adjusted to pH 9.5 using hydrochloric acid or sodium hydroxide diluted solution. 100 mM of sucrose solution was prepared by dissolving sucrose (0.274 g) in MilliQ water (8 mL) and 100 mM glucose solution was prepared by dissolving glucose (0.270 g) in borax buffer (15 mL).

- - 1. **Film hydration method to get the GUV**

The phospholipid solutions were prepared by mixing DOPC (1 mg.mL^-1^ in CHCl_3_, 9.8 µL), DSPE-PEG-Biotin (1 mg.mL^-1^ in CHCl_3_, 0.1 µL) and liss rhod PE (1 mg.mL^-1^ in CHCl_3_, 0.1 µL) in CHCl_3_. PVA solution (20 µL) was deposited onto a glass coverslip and was dried in an oven at 80°C for 30 minutes. Adequate phospholipids solution (10 µL) was dropped onto the resulting dried PVA layer, the obtained phospholipid film was vacuumed for 30 minutes. A well was built manually all around the phospholipids and PVA deposition area using a hematocrit closure wax VITREX (VMG, Denmark), and sucrose solution (200 µL) was added within the well. The well was sealed with a 6x6 glass coverslip. The GUVs were allowed to form and swell for at least 3 hours. In an 1 mL Eppendorf tube **AP-Strep** solution was added 2 µL, 1 mg.mL^-1^ in borax buffer) (when needed) and borax buffer (200 µL). 100 µL of the GUV solution was collected from the glass coverslip with a micropipette and added to the previous mixture. GUV were allowed to sediment for at least one hour before use.

## Preparation of SUV/AP-Strep

**1.3.1. Sonication extrusion method**

DOPC (4.95 mg) was added to a 5 mL round bottom flask, DSPE-PEG-Biotin was added (5 µL, 10 mg.mL^-1^ in CHCl_3_). Chloroform was added to fully solubilize the mixture. The obtained solution was dried under reduced pressure (rotary evaporator). MilliQ water (1 mL) was added, and the resulting suspension was submitted to sonication for 3 min. The obtained suspension was then filtered through a 0.8 µm membrane. 21 passages were performed. The so-filtered solution was submitted once again to sonication for 3 min. 10%-biotin SUVs were prepared following the exact same method using DOPC (4.5 mg) and DSPE-PEG-Biotin (50 µL, 10 mg.mL^-1^ in CHCl_3_). **SUV/Biotin** solutions can be stored at 4°C.

- - 1. **Addition of AP-Strep and Sepharose column purification of SUV/AP-Strep**

AP-Strep (5 µL, 1 mg.mL^-1^ in borax buffer) was added to **SUV/Biotin** solution (200 µL) prepared as described just above. To remove the excess of AP-Strep, Sepharose column chromatography was performed. Sepharose CL-4B was poured into a column with a 0.7 cm inner diameter, until an 8 cm height was reached. Equilibration was performed with borax buffer on five column volumes at a flow rate of 1 mL.min^‑1^, using a peristatic pump (see picture below). Blue dextran solution (1 mg.mL^-1^, 200 µL) was used to determine the dead volume of the column, and also to determine when the **SUV/AP-Strep** would come out of the column. The eluent coming out of the column was collected in Eppendorf tubes (250 µL/tube), the ones having a meniscus contained **SUV/Biotin** (see below).


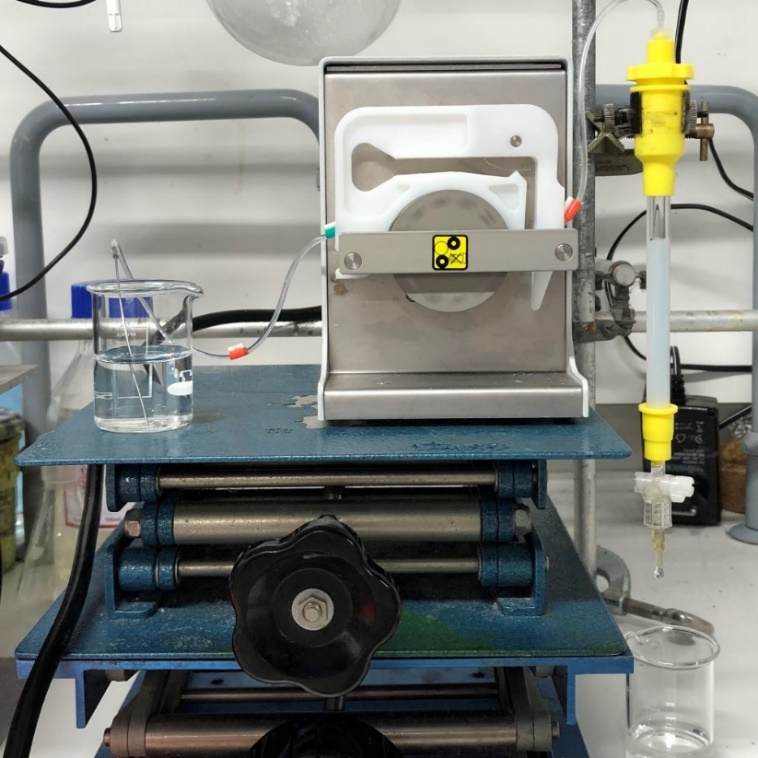

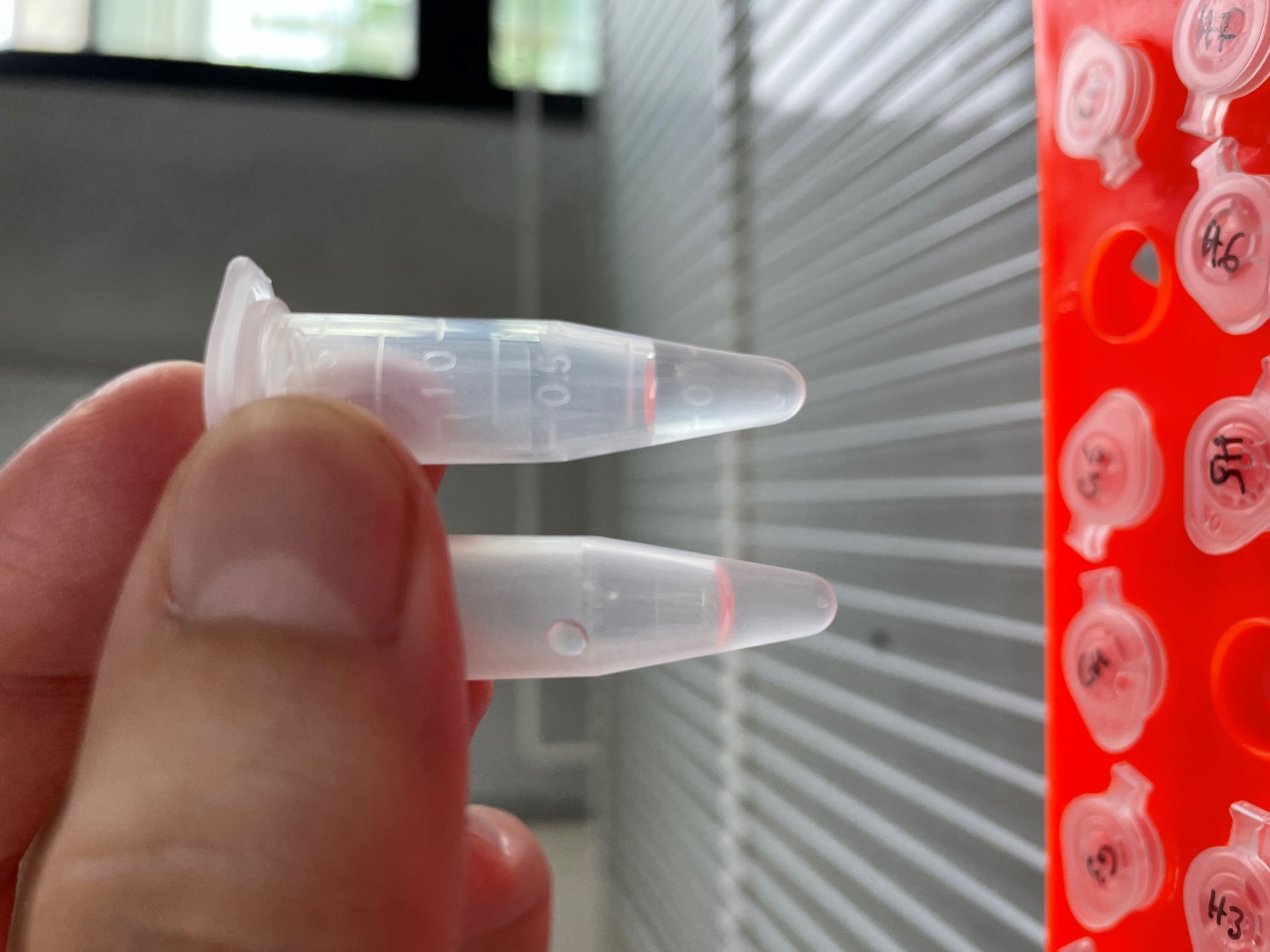


**b**

**iv**

**v**

**iii**

**ii**

**i**

**a**

(**a**)Picture of the experimental set up with (**i**) borax buffer, and a (**ii***)* peristatic pump connected to the (**iii***)* Sepharose column. (**b**) Solution containing **SUV/AP-Strep**, presence of a meniscus (**iv**) and (**v**) a solution without **SUV/AP-Strep** used as reference.

- 1. **Phase-contrast and epifluorescence microscopy**

Phase contrast and epifluorescence microscopy observations were performed using a Nikon ECLIPSE TE200 microscope with a 40x optical lens (unless stated otherwise). Images were captured using a BFI Optilas camera from DIGANOSTICS Instrument. Rhodamine was excited with an excitation filter at 561 nm, and ThT at 458 nm. The images were acquired in shades of grey and reworked using ImageJ software, with rhodamine fluorescence coloured red and ThT fluorescence coloured green. The transition from phase contrast to epifluorescence mode was achieved by simply manipulating levers, which allowed an excitation filter to be added and the annular diaphragm to be removed from the optical path.

- 1. **Confocal laser scanning microscopy**

Samples were prepared the same way as described for the epifluorescence microscopy and images were captured using ZEISS LSM 710. ThT was excited at 458 nm, Rhod-B was excited at 561 nm. Raw images were in shades of gray, ThT was colorized in green and Rhod-B in red.

- 1. **Cryo-Transmission electron microscopy**

5 µL of the sample mixture was deposited onto a freshly glow discharged holey carbon film-covered Cu-grid (400 mesh). Rapidly frozen by plunging into a liquid ethane bath cooled by liquid nitrogen in a home-made freezing machine (controlled T°=22°C and RH>80%). The grid is transferred onto a Gatan 626 cryo holder and finally observed in a 200kV Tecnai G2 microscope (FEI) under low dose conditions. Images were acquired with a camera Eagle 2k (FEI) ssCD camera.

- 1. **Molecular dynamic simulations**

Classical molecular dynamics (cMD) simulations were performed using the **AMBER18** software suite.**^[ref 1]^** The potential energy, U, was described as the sum of bond, angle, and dihedral deformation energies, along with pairwise additive electrostatic and van der Waals terms between nonbonded atoms (eq 1):


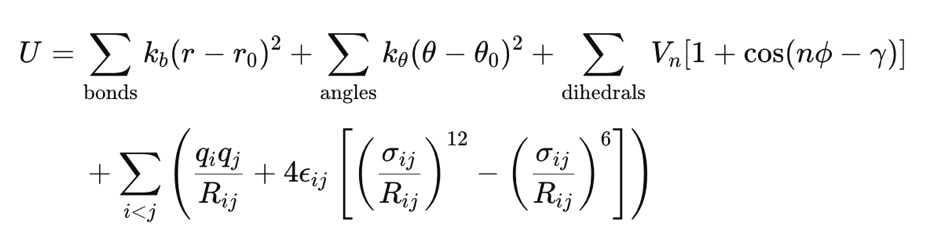


*(1)*

During the cMD simulation we used a truncated octahedron box of 129.9 Å side length. Inside this box we randomly placed 75 Fmoc-FFY peptides around an AP protein together with 48807 water molecules. Additionally, we added 195 Na^+^ cations and 100 Cl^-^ anions in order to neutralize the system. Force field parameters for the AP protein and Fmoc-FFY peptide were taken from the AMBER ff14SB force field,**^[ref 2]^** while those for the Na⁺ and Cl⁻ are taken from the work of Cheatham et al. **^[ref 3]^** Water molecules were described using the TIP3P model. **^[ref 4]^** Atomic charges for the Fmoc fragment were obtained via the RESP procedure.**^[ref 5]^** Cross terms in van der Waals interactions were constructed using the Lorentz–Berthelot combining rules. The 1–4 van der Waals and 1–4 electrostatic interactions were divided by factors of 2 and 1.2, respectively. 3D periodic boundary conditions were applied to the system. An atom-based cutoff of 9 Å was applied for electrostatic and van der Waals interactions, and long-range electrostatics were computed using the particle mesh Ewald (PME) method. After equilibration, production cMD simulations of 1 000 ns were performed in the NVT ensemble. The temperature was maintained at 298.15 K using a Berendsen thermostat with a relaxation time of 1.0 ps.**^[ref 6]^** The equations of motion were integrated using a 1 fs time step and coordinates of the system were saved every 5 ps. Snapshots along the trajectory were analyzed and visualized using the VMD software. **^[ref 7]^** For the determination of the existence of a hydrogen bond between the AP protein and the Fmoc-FFY peptide we used the following definition: A hydrogen bond is formed between an atom with a hydrogen bonded to it (the donor, D) and another atom (the acceptor, A) provided that the distance D-A is less than the cut-off distance of 3.0 Å and the angle D-H-A is less than the cut-off angle of 20 degrees.

**^[ref 1]^** D.A. Case, I.Y. Ben-Shalom, S.R. Brozell, D.S. Cerutti, T.E. Cheatham, III, V.W.D. Cruzeiro, T.A. Darden, R.E. Duke, D. Ghoreishi, M.K. Gilson, H. Gohlke, A.W. Goetz, D. Greene, R Harris, N. Homeyer, Y. Huang, S. Izadi, A. Kovalenko, T. Kurtzman, T.S. Lee, S. LeGrand, P. Li, C. Lin, J. Liu, T. Luchko, R. Luo, D.J. Mermelstein, K.M. Merz, Y. Miao, G. Monard, C. Nguyen, H. Nguyen, I. Omelyan, A. Onufriev, F. Pan, R. Qi, D.R. Roe, A. Roitberg, C. Sagui, S. Schott-Verdugo, J. Shen, C.L. Simmerling, J. Smith, R. Salomon-Ferrer, J. Swails, R.C. Walker, J. Wang, H. Wei, R.M. Wolf, X. Wu, L. Xiao, D.M. York and P.A. Kollman (**2018**), AMBER 2018, University of California, San Francisco.

**^[ref 2]^** Maier, J. A.; Martinez, C.; Kasavajhala, K.; Wickstrom, L.; Hauser, K.E.; Simmerling, C. “ff14SB: Improving the accuracy of Protein Side Chain and Backbone Parameters from ff99SB.” J. Chem. Theory Comput., **2015**, *11*, 3696−3713.

**^[ref 3]^** Joung, I. S.; Cheatham, T. E. “Determination of Alkali and Halide Monovalent Ion Parameters for Use in Explicitly Solvated Biomolecular Simulations.” J. Phys. Chem. B, **2008**, *112*, 9020−9041.

**^[ref 4]^** Jorgensen, W. L.; Chandrasekhar, J.; Madura, J. D.; Impey, R.W.; Klein, M. L. “Comparison of Simple Potential Functions for Simulating Liquid Water.” J. Chem. Phys.,**1983**, *79*, 926−935.

**^[ref 5]^** Bayly, C. I.; Cieplak, P.; Cornell, W. D.; Kollman, P. A. “A Well Behaved Electrostatic Potential Based Method Using Charge Restraints for Deriving Atomic Charges: The RESP Model.” J. Phys. Chem., **1993**, *97*, 10269−10280.

**^[ref 6]^** Berendsen, H. J. C.; Postma, J. P. M.; Van Gunsteren, W. F.; Dinola, A.; Haak, J. R. “Molecular Dynamics with Coupling to an External Bath.” J. Chem. Phys., **1984**, *81*, 3684−3690.

**^[ref 7]^** Humphrey, W.; Dalke, A.; Schulten, K. “VMD: Visual Molecular Dynamics.” J. Mol.Graph., **1996**, *14*, 33−38.

**2. Supplementary Figures**

**Figure S1.** Typical phase-contrast microscopy images of (*left*) **GUV/AP-Strep** observed at t = 24h, (*middle*) **GUV-Biotin** in presence of Fmoc-FF*p*Y (0.1 mg.mL^-1^) at t = 24h, and (*right*) **GUV-Biotin** in presence of Fmoc-FF*p*Y (0.1 mg.mL^-1^) and AP (5 µL, 1 mg.mL^-1^, borax buffer) at t = 24h.

| **GUV/AP-Strep**  t = 24h | **GUV-Biotin** + FmocFF*p*Y  t = 24h | **GUV-Biotin** + FmocFF*p*Y + AP free in solution  t = 24h |
| --- | --- | --- |
| 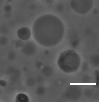 | 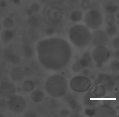 | 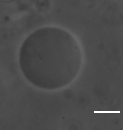 |
| scale bar = 10 µm | | |

**Figure S2.** Typical cryo-TEM images of **SUV/AP-Strep** brought in contact with Fmoc-FF*p*Y solution (10 mg/mL) after 24h.


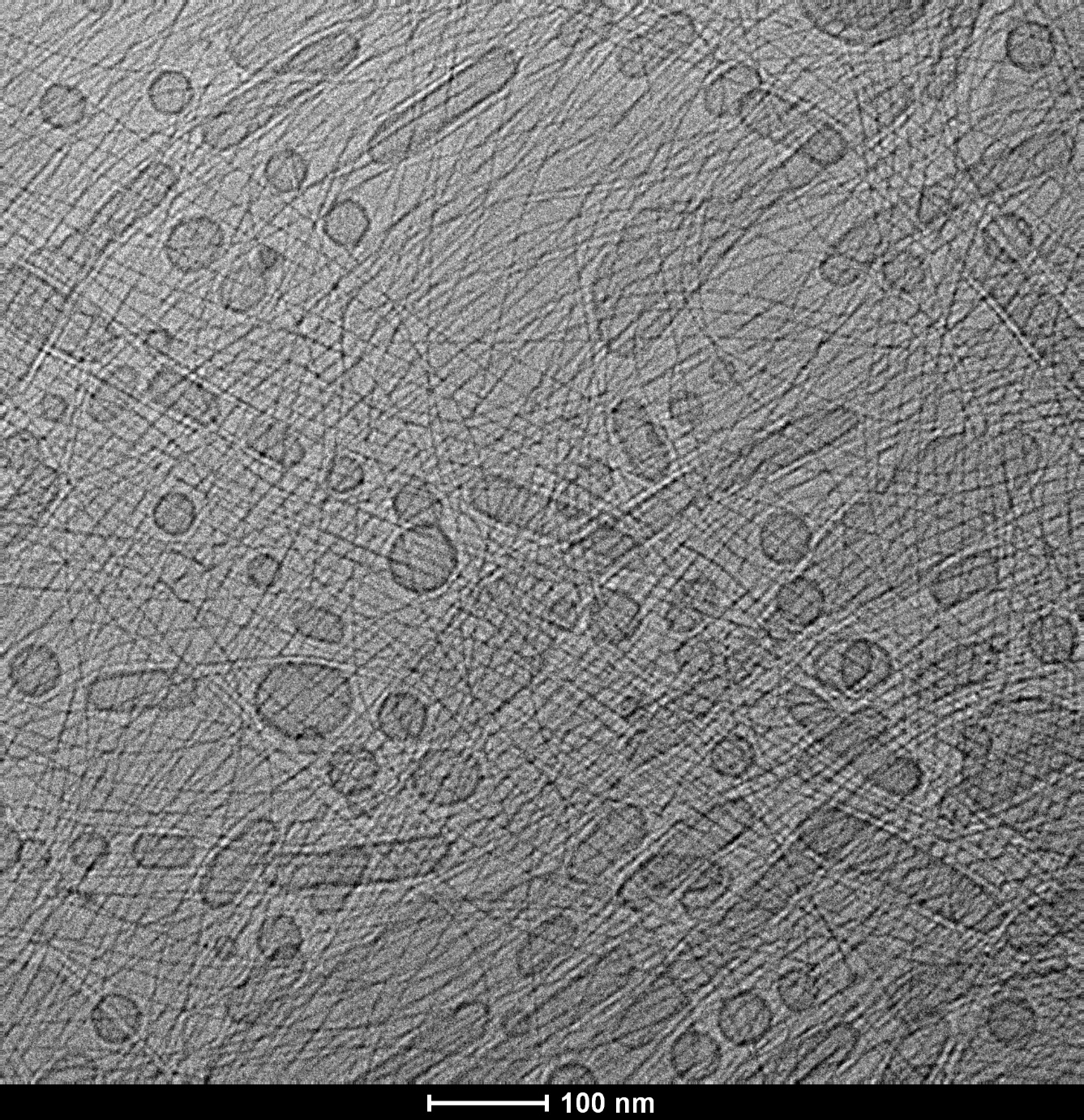


**Figure S3.** Evolution of the number of hydrogen bonds (H-Bonds) number over time (from 0 to 1 µs, with a time step of 1.25 ns). Black line: total number of hydrogen bonds between AP and Fmoc-FFY; green line: hydrogen bonds in which AP acts solely as an H-bond donor (HBD); red line: hydrogen bonds in which AP acts as an H-bond acceptor (HBA).


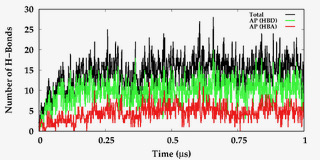


**Figure S4.** (*left*) Zoomed-in view of the LYS (AP) ↔ TYR (Fmoc-FFY) interaction observed in molecular dynamics simulations. Carbon atoms shown in green correspond to AP moieties, while carbon atoms shown in red correspond to the Fmoc-FFY peptides. (*right*) Zoomed-in view of the ARG (AP) ↔ TYR (Fmoc-FFY) interaction observed in molecular dynamics simulations. Carbon atoms shown in green correspond to AP moieties, while carbon atoms shown in red correspond to the Fmoc-FFY peptides.

**
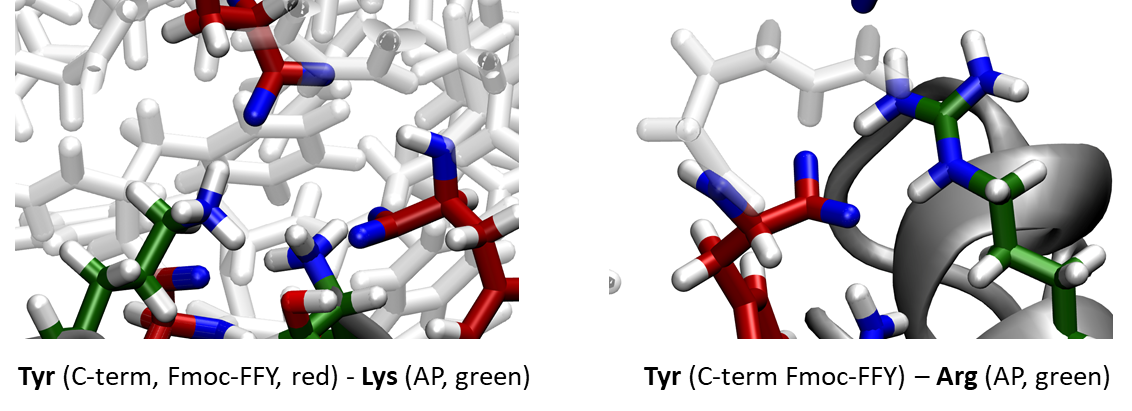
**
